# Epidermal Stem Cells in Wound Healing and Regeneration

**DOI:** 10.1155/2020/9148310

**Published:** 2020-01-29

**Authors:** Ronghua Yang, Jingru Wang, Xiaodong Chen, Yan Shi, Julin Xie

**Affiliations:** ^1^Department of Burn Surgery, The First People's Hospital of Foshan, Foshan 528000, China; ^2^Department of Medical Cosmetology, Jiangxi Maternal and Child Health Hospital, Nanchang 330006, China; ^3^Department of Burn Surgery, First Affiliated Hospital of Sun Yat-Sen University, Guangzhou 512100, China

## Abstract

Skin stem cells distributed in the basal layer of the epidermis and hair follicles are important cell sources for skin development, metabolism, and injury repair. At present, great progress has been made in the study of epidermal stem cells at the cellular and molecular levels. Stem cell transplantation is reported to promote skin healing, endothelial cell transformation, and vascular formation. Local stem cells can also be transformed into keratinocytes, sebaceous gland, and other skin-associated tissues. However, the mechanism of action of epidermal stem cells on wound healing and regeneration is not completely clear. This review is aimed at briefly summarizing the biological characteristics of epidermal stem cells and their clinical application in wound healing and tissue regeneration. It further discussed the mechanism of action and the development direction in the future.

## 1. Introduction

The strong repair and regenerative abilities of skin is inextricably related to the presence of stem cells. At present, no consensus has been reached regarding the type, density, and function of skin stem cells. However, it has been proved that skin stem cells (predominantly epidermal stem cells (EpiSCs) and hair follicle stem cells) distributed in the basal layer of the epidermis and the hair follicle bulge are important sources of cells for regeneration, metabolism, and wound repair of skin. The hair follicle stem cells can not only participate in the morphogenesis of hair follicles but also play a critical role in wound healing [1, 2]. Generally, the more the residual skin stem cells on the wound surface, the faster the healing speed, and the less the scar formation. For example, the scalp is often used as a skin donor site in clinical practice, because it contains a considerable amount of stem cells, which can be regenerated and repaired. For deep wounds, scar hyperplasia inevitably occurs after healing due to the loss of most of the skin stem cells [3]. The normal human skin has a variety of stem cells with multilineage differentiation potentials. Theoretically, any wound can rely on skin stem cells to achieve physiological healing. However, for deep wounds occurring in skin appendages, the residual skin stem cells cannot undergo normal differentiation and complete the anatomical structure and functional skin repair according to the preprogrammed pathways; as a result, the healing process may be out of control, eventually forming scar tissues without hair and sweat glands [3, 4]. This indicates that the outcome of wound healing is not only related to the number of skin stem cells but also their differentiation behaviors.

The stem cells have been found to have remarkable efficacy in animal experiments and clinical studies of many diseases due to their multilineage differentiation potentials, anti-inflammatory, paracrine, and other biological functions [5]. In recent years, the application of various types of stem cells in treating wounds has been increasingly appreciated by many scholars. The systemic or local implantation of stem cells in refractory wound surfaces of animals can contribute to wound healing [6–8], as the transplanted stem cells have potential to differentiate into keratinocytes, sebaceous glands, and other skin appendages [9]. The process wound healing is related to interaction between cells, complicated regulation of the extracellular matrix, and miscellaneous paracrine factors. Stem cells had a therapeutic effect on wound healing; however, the mechanism of action of EpiSCs on wound healing is poorly understood [10]. The objective of the present review was to further investigate the characteristics of EpiSCs and their clinical application, mechanism, outlooking wound healing, and tissue regeneration.

## 2. Characteristics of EpiSCs

### 2.1. Concept of EpiSCs

EpiSCs are located mainly in the basal layer and hair follicle bulge with the rich blood supply in the epidermis. They are a cell population with infinite proliferative potential that can achieve self-renewal through symmetric or asymmetric division, continuously producing functional cells to maintain the homeostasis of the epidermis. Morphologically, EpiSCs have the characteristics of undifferentiated cells: relatively small cell volume, large nucleus with less cytoplasm, high nuclear to cytoplasmic fluorescence ratio, low intracellular RNA content, fewer and immature organelles, and relatively stable position in tissue structure [11]. In 1981, Bickenbach [12] first used tritiated thymine nucleotides (3H-TdR) and found a cell population in which a marker had a retention time of up to 2 years in mouse basal-layer cells, which was later confirmed as EpiSC. The division mode of EpiSC is mainly asymmetric. Each time stem cells divide, two daughter cells can be produced. One has the characteristics of stem cells themselves, and the other one differentiates into transient amplifying cells (TACs). The latter divide three to five times forming terminally differentiated cells (TDCs) [13, 14].

### 2.2. Characteristics of EpiSCs

EpiSCs are adult stem cells with three typical characteristics: (1) stronger self-renewal ability, (2) slow cycling, and (3) adhesion to the basement membrane of the epidermis. EpiSCs adhere mainly to the basement membrane via the expression of integrin, which is essential for maintaining stability in the basal layer and also important for establishing the spatial distribution of the skin appendages [13].

### 2.3. Sources of EpiSCs

The sources of EpiSCs may be as follows: (1) storage in the basal layer with rich blood supply in the epidermis; (2) stem cells in the hair follicles, which are the warehouse of EpiSCs; (3) reverse production from differentiated cells; (4) regeneration of mesenchymal cells into EpiSCs; (5) induced differentiation of embryonic stem cells; and (6) migration of hematopoietic stem cells or other tissue stem cells to the skin tissue with the blood circulation under the stimulation of certain factors, which transversally differentiate into EpiSCs due to the plasticity of stem cells.

### 2.4. Markers of EpiSCs

Although EpiSCs have the self-renewal and multilineage differentiation potentials, more efforts should still be made regarding the effective separation of EpiSCs. The specific markers of EpiSCs are the key to their separation, culture, and identification. Many markers are now used to identify EpiSCs, but no markers have been found that can separate EpiSCs at the single-cell level. Nowadays, the more commonly used specific markers for identifying EpiSCs include cell surface glycoproteins (such as integrin), keratin, and nucleoprotein p63.

#### 2.4.1. Integrin

Integrin is a family of glycoprotein receptors located on the surface of cell membranes, which integrates the cytoskeleton with the extracellular matrix by mediating the adhesion between cells and the extracellular matrix. In addition, it is also involved in the cell-to-cell adhesion [15]. As an important type of adhesion molecule, integrin involves many important pathophysiological processes and plays a key role in cell division, differentiation, apoptosis, inflammatory response, tissue repair, tumor invasion, and metastasis. Integrin comprises one *α* subunit and one *β* subunit; different *α* and *β* subunits form a variety of different integrins.

Among them, the integrin composed of the *β*1 subunit plays an important role in the adhesion of EpiSCs to the basement membrane. EpiSCs adhere mainly to the basement membrane via the expression of integrin, and EpiSCs in the basal layer of the skin adhere to the basement membrane and the extracellular matrix through the integrin on the surface. Hence, the EpiSCs are the cells with the highest expression level of integrin. During the process of proliferation and differentiation of basal-layer cells, the expression of the surface integrin is gradually downregulated until it disappears, and the cells gradually migrate to the surface of the skin and are finally keratinized and exfoliated. Studies have demonstrated [16–18] that the decrease in *β*1 integrin expression stimulates hair follicle stem cells to leave the stem cell pool and migrate upward into differentiated cells [19, 20]. Moreover, some researchers have found that nitric oxide (NO) can mediate the regulation of *β*1 integrin expression, affect the proliferation and differentiation of EpiSCs through cGMP signaling pathway, and then participate in the process of wound healing [21]. Given that *β*1 integrin is highly expressed on the surfaces of EpiSCs and TACs, and TDCs have low or no expression of *β*1 integrin, the antibodies of *β*1 integrin can be used to identify EpiSCs and TACs. Although the expression level of surface *β*1 integrin of EpiSCs is more than twice that of TACs, it is still impossible to distinguish between EpiSCs and TACs according to the difference in the positive expression intensity of *β*1 integrin under a light microscope. However, if the laser confocal microscope is used to observe at the level of monolayer cells, or flow cytometry and fluorescence-activated cell separation are used in combination, the difference in the positive expression intensity of *β*1 integrin between the two cells can be identified, thus achieving the identification of EpiSCs and TACs [22, 23]. Recent studies have found that long noncoding RNA (lncRNA) expression near the *β*1 integrin is regulated by *β*1 integrin and epidermal growth factor (EGF) signaling pathway, and the downregulation of the gene expression marks the transformation of EpiSCs from proliferation to differentiation [24].

In contrast, the high expression of *α*6-integrin and low expression of transferrin receptor (*α*6-bright/CD71-dim) are widely recognized means for identifying EpiSCs to date [25, 26]. The *α*6-integrin is present on the lower lateral surface of the basal cells through which the cells adhere to the basement membrane. *α*6briCD71dim cells are relatively stationary in the body, but these cell populations have high long-term proliferative capacity [27]. Researchers detected these cells in keratinocytes on the back of mice and found that they were similar to the epithelial stem cells in characteristics: small cell populations with a high nuclear to cytoplasmic ratio [26, 28]. In addition, they found that the proportion of these cells in keratinocytes was 1.4% [29]. Moreover, *α*6briCD71dim cells have also been found in human skin. They are small cell populations with a high nuclear to cytoplasmic ratio and still have the ability to produce a large number of cell populations after 10 days of *in vitro* culture. At the same time, *α*6briCD71dim cells can induce differentiation to produce multilayered and thick epidermal tissues, while *α*6briCD71bri cells differentiate into thin skin tissues with disorganized stratification [30].

#### 2.4.2. Keratin

Keratin is an important structural protein of epidermal cells. The different degrees of differentiation of epidermal cells can be used to express different characteristics of keratin, which can be used for identifying EpiSCs, TACs, and TDCs [31]. EpiSCs express mainly keratin 15 and 19 (CK15 and CK19); TACs express keratin 5 and 14 (CK5 and CK14); and TDCs express keratin 1 and 10 (CK1 and CK10). Some scholars [32, 33] conducted double-labeling experiments, showing that cells with positive expression of CK19 had the following characteristics: (1) cells with positive expression of CK19 were (3H) label-retaining cells with slow-cycle characteristics of stem cells and (2) the expression of *β*1 integrin for cells with positive expression of CK19 was also positive, indicating that CK19 could be used as a specific marker for EpiSCs [34, 35]. Lyle et al. [36] found that EpiSCs in the hair follicle bulge showed high expression of CK15, and in the process of EpiSCs differentiation, the decrease in CK15 expression occurred earlier than the decrease in CK19 expression. Presumably CK15-negative and CK19-positive cells might be “early” TACs, and thus the use of CK15 in combination with CK19 to identify EpiSCs was more meaningful [37, 38]. In addition, recent studies have shown that the targeted inhibition of CK15 expression can promote the expression of miR-184, thereby inhibiting the proliferation of EpiSCs and promoting differentiation [39]. Julin et al. [40] found that EpiSCs transform into TDCs and express CK10 under physiological and pathological conditions. Therefore, CK10 is a characteristic protein of epidermal cells with a high degree of markedness and differentiation. The use of CK10 as a negative molecular marker for EpiSCs and TACs is of importance for sorting and identifying EpiSCs.

#### 2.4.3. Nucleoprotein p63

Nucleoprotein p63 is a homologous gene transcription factor of p53, and its structure and function are similar to those of p53. At least six protein subtypes of p63 exist, and these are divided into two groups based on transcription activation (TA): TA subtype (TA-p63*α*, TA-p63*β*, and TA-p63*γ*) and N-TA subtype (*Δ*N-p63*α*, *Δ*N-p63*β*, and *Δ*N-p63*γ*) [21]. Both TA subtype and p53 are similar in activating the transcription of specific target genes and inducing cell cycle arrest and apoptosis, while N-TA subtype cannot activate transcription [41]. The nucleoprotein p63 is widely and selectively expressed in a variety of human tissues, especially in proliferating epithelial tissues [42]. Studies have shown an important role of p63 in skin development, maintenance of an undifferentiated state of stem cells, and stem cell proliferation [43]. Researchers used immunofluorescence to confirm the use of p63 as a marker for EpiSCs [44]. Pellegrini et al. [45] also demonstrated that, at the molecular level, nucleoprotein p63 could be specifically expressed in EpiSCs and used to distinguish between EpiSCs and TACs. In addition, researchers have recently introduced the concept of histone deacetylases (HDACs), suggesting that the positive expression of p63 and negative expression of HDAC may be an effective new means for identifying EpiSCs [46].

## 3. Regulation Mechanism of EpiSCs Proliferation and Differentiation and Its Relationship with Wound Repair

Despite in-depth studies regarding the phenotypic characteristics, functions, and roles of EpiSCs, the specific regulation mechanism of proliferation and differentiation remains poorly understood. The microenvironment of stem cells, also known as “stem cell niches,” plays a key role in regulating the migration, proliferation, and differentiation of stem cells, and this effect is achieved by a network system of multiple intertwined signaling pathways [47]. Among them, wingless/integrated (Wnt) and Notch signaling pathways are important components of stem cell “niches” that play an important role in skin development and wound repair [48–50]. When the skin is damaged, the microenvironments that affect stem cell differentiation, such as the changes in the number of repair cells, concentration of cytokines, and components of the extracellular matrix (ECM), result in the activation of the regulatory network, including Wnt and Notch signaling pathways in the wound tissues [51]. Thus, the differentiation and proliferation of wound EpiSCs are induced, eventually contributing to wound healing or scar formation. However, how the signaling pathway regulates the proliferation and differentiation of EpiSCs and its association with wound repair and scar formation remain unclear.

### 3.1. Regulation of the Effect of Wnt Signaling Pathway on the Differentiation of EpiSCs

Wnt signaling protein is a secreted glycoprotein that plays an important role in maintaining metabolic homeostasis [52]. It can regulate the proliferation, differentiation, and migration of related cells [53, 54]. At present, related studies suggest that Wnt signaling protein reaches the target cells mainly through diffusion and active transport and binds to the transmembrane receptor Frizzled protein family (Frz) or low-density lipoprotein receptor-related protein family on the surface of target cells, causing the accumulation of the second messenger *β*-catenin in the cytoplasm and thereby activating the cascade reaction of downstream components [55]. The key switch in the canonical Wnt pathway is the cytoplasmic protein *β*-catenin. It is an essential binding partner for the cytoplasmic tail of various cadherins, such as E-cadherin in adhesion junctions. While the half-life of the signaling pool of *β*-catenin is in the order of minutes, the adherens junction pool is highly stable. Its stability is controlled by the destruction complex [54]. When the Wnt signal is “OFF,” the second messenger *β*-catenin is phosphorylated after binding to a polyprotein complex (including APC, Axin, and glycogen synthase kinase-3*β* (GSK-3*β*)), which is then ubiquitinated and degraded. GSK-3*β*, a serine-threonine protein kinase, is involved in regulating the stability of *β*-catenin and plays a key role in the destruction complex [56]. The binding of the Wnt protein to the transmembrane receptor blocks GSK-3*β*-mediated phosphorylation of *β*-catenin, resulting in the accumulation of *β*-catenin in the cytoplasm. Subsequently, it enters the nucleus to bind to T-cell factor/lymphoid enhancer factor (TCF/LEF) transcription factor family members, activating the transcription of the target genes (c-Myc, cyclin D1, and so forth), thus activating this signaling pathway [49].

Recent studies have shown that the Wnt signaling pathway is closely related to skin wound repair and scar formation. EpiSC proliferation is limited when the *β*-catenin expression is specifically downregulated [57–59]. Further studies have demonstrated that the inhibition of the activity of the Wnt signaling pathway by DKK1 can also reverse the excessive proliferation of EpiSCs [60, 61]. On the contrary, researchers found that the high expression of the Wnt signaling pathway is present in epidermal cells during the embryonic period [62]. At the same time, cells with high expression of the Wnt signaling pathway in the basal layer of the skin have characteristics of the slow cell cycle. They can form transient progenitor cells and eventually differentiate into other cells, while cells with low expression of the Wnt signaling pathway in the upper basal part do not have the aforementioned abilities [63]. A high level of Wnt signaling can induce stem cells to develop into structures of hair and sebaceous gland, while blocking Wnt signaling leads to the differentiation of EpiSCs in the epidermis [64]. During the repair of skin wounds, the Wnt/*β*-catenin signaling pathway is activated, and the wound surface is covered by the stratified epithelium. Moreover, the formation of simple appendages, such as hair follicles and sebaceous glands, can be observed. No aggregation of inflammatory cells and the deposition of many collagens occur in the dermis after repair. Hence, activating the Wnt/*β*-catenin signaling pathway can significantly improve the quality of skin healing [65]. Therefore, it is hypothesized that changes in Wnt/*β*-catenin signaling pathways may be one of the important molecular mechanisms of abnormal wound healing and scar formation in deep skin damage, and the intervention of signaling pathways to regulate the differentiation of EpiSCs may improve healing quality.

### 3.2. Regulation of the Effect of Notch Signaling Pathway on the Differentiation of EpiSCs

The Notch signaling pathway is an evolutionarily highly conserved signaling pathway, which is present in signal transduction systems that determine cell fate in a variety of tissues [66, 67]. This signaling pathway comprises Notch receptor protein (Notch1-4), Notch ligand protein of adjacent cells (Jag1, Jag2, and so forth), and DNA-binding protein [68]. When the extracellular domain of Notch binds to the ligand, the Notch receptor protein undergoes three steps of cleavage after an enzymolysis process involving the *γ*-secretase, releases the activated form of the Notch intracellular domain (NICD), and then enters the nucleus to bind to the CSL (CBF1, Suppressor of Hairless, Lag-1), a DNA-binding protein, to activate the expression of the target genes [69]. The known target genes include mainly hairy and enhancer of split (HES) and HRT/HERP, wherein the HES protein expressed by the target gene is a transcriptional repressor [70]. The continuous activation of the Notch signaling pathway can upregulate the transcription of the HES1 gene, thereby inhibiting the transcription of the target gene [71]. The latter is involved in regulation or synergizes with other signaling pathways to regulate cell proliferation, differentiation, migration, and apoptosis [72]. The Notch receptor must be hydrolyzed and cleaved by the protease *γ*-secretase to release the intracellular domain, thereby activating the signaling pathway. 4,6-diamidino-2-phenylindoleindole (DAPT), as a blocker of the Notch signaling pathway, can specifically block the action of *γ*-secretase, thereby preventing the activation of Notch [73].

The Notch signaling pathway plays a decisive role in regulating the proliferation and directional differentiation of pluripotent stem cells (PSCs) [74]. After the Notch receptor binds to its ligand Jag protein, the stem cells can maintain multilineage differentiation potentials for nondifferentiated proliferation, and when the activity of Notch signaling is inhibited, the stem cells are susceptible to differentiate into TDCs [74, 75]. Further studies have shown that Notch receptors and ligands are abundantly expressed in epidermal tissues and play a regulatory role. Meanwhile, the evolutionarily conserved Notch pathway is widely used in mammalian embryonic development and mature tissues to determine cell fate and plays a key role in regulating the proliferation and differentiation of epidermal tissue cells [72, 76]. Studies have demonstrated that Notch1 is widely expressed [77] while EpiSCs are located mainly in the basal layer of the epidermis. In addition, after specifically knocking out Jag1, basal-layer cells lost their proliferative capacity and differentiated abnormally [76, 78]. However, the low specific expression of Hes1 leads to a limited proliferation of skin EpiSCs, impeding the epithelialization process. Thus, skin barrier function damage and abnormal hair development occur [79, 80]. One of the explanations is that the Notch signaling pathway can regulate stem cell adhesion; the latter affects the biological microenvironment (“niche”) of stem cells. Consequently, the stem cells receive different signals and self-renew or differentiate along different lineages [81]. Of note, Jag1, as the first confirmed ligand of Notch receptor in mammals, is expressed in the whole layer of skin. It plays an important role in regulating the differentiation of EpiSCs [82, 83]. Recent studies found that Jag1 mediated the “dialogue” of Notch and Wnt signaling in skin tissue, thereby regulating EpiSC proliferation and differentiation and playing a role in wound healing and scar formation. However, the specific mechanism remains unclear, and further research is needed.

### 3.3. Regulation of the Effect of MicroRNAs on EpiSCs and Wound Healing

MicroRNAs (miRNAs) are small RNAs that regulate the expression of complementary messenger RNAs. In the nucleus, the pri-miRNA is processed into a 70- to 100-nt stem-loop pre-miRNA (pre-miRNA) by the RNase III enzyme Drosha, which is subsequently transported to the cytoplasm by exportin-5 and processed into 21- to 23-nt mature miRNA via another RNase III enzyme Dicer. Its mechanism of action is as follows: the mature miRNA base pair with the 3′-terminal untranslated region (UTR) of the target mRNA, forming an RNA-induced silencing complex and thereby negatively regulating the expression of related target genes [84, 85]. In fact, one miRNA can be involved in the regulation of hundreds of genes, while one gene can be regulated by multiple miRNAs [86]. Therefore, miRNAs are key factors in the regulation of gene expression and play a key role in many biological processes, including cell proliferation and differentiation [85, 87].

In recent years, the roles of miRNAs in the development of epidermal tissue and the maintenance of the homeostasis of adult skin stem cells have also attracted increasing attention [88–90]. The specific expression of several miRNAs has been detected in different epidermal tissue components of mice [91]. For example, miR-200 (a, b, and c), miR-141, miR-429, miR-19b, and miR-20 are expressed in the whole layer of epidermal tissue, while the expression of the miR-199 family can only be detected in hair follicles [90, 91]. Hildebrand et al. found that the expression of miR-203, miR-23b, miR-95, miR-210, miR-224, miR-26a, miR-200a, miR-27b, and miR-328 was upregulated in differentiated keratinocytes, while the expression of miR-376a decreased [92], indicating the involvement of the aforementioned upregulated miRNAs in the differentiation of epidermal cells. miR-203 plays a role in maintaining the morphology of skin tissues and differentiation of keratinocytes through inhibiting the expression of p63 and leading to the loss of characteristics of EpiSCs and premature differentiation [93–95]. However, during wound healing, the overexpression of miR-203 can ultimately lead to a decrease in the number of EpiSCs, skin barrier defects, and so forth. The expression of miR-203 was downregulated in the wound edges of acute skin wounds, while the expression of p63 was upregulated. However, the expression of miR-203 was upregulated in chronic wounds [96, 97]. Moreover, related studies have also found an important regulatory role of miR-125b in the biological activities of EpiSCs; increased expression of miR-125b can promote EpiSCs to maintain their characteristics, while the expression of miR-125b decreases in the daughter cells of EpiSCs [98]. In addition, uncontrolled expression of miR-125b is an important cause of excessive proliferation of epidermal cells in patients with psoriasis.

Increasing evidence shows that miRNAs are stably present in body fluids, including saliva, urine, breast milk, and blood, and are involved in the circulation of exosomes [99]. These exosomes can protect miRNAs from degradation and stabilize them. miRNAs transmit information through exosomes, which is considered to be the third pathway for intercellular signaling, as important as the other two pathways: cellular contact-dependent signal transduction and soluble molecular-mediated conduction [100–102]. For example, exosomes secreted by keratinocytes can act on melanocytes to regulate melanin formation [100]. Mistry et al. found that some exosome subunits were abundantly expressed in epidermal progenitor cells, which could maintain the proliferation of related cells and inhibit the premature differentiation of cells [103]. The aforementioned studies undoubtedly provide new insights and directions for studying the regulation of EpiSCs by miRNA.

### 3.4. Regulation of the Effect of lncRNA on EpiSCs and Wound Healing

In the recent two decades, related studies have revealed important regulatory roles of many noncoding RNAs (ncRNAs) in cell physiology and pathology [33]. lncRNA is an ncRNA with more than 200 nucleotides that contains numerous and diverse ncRNA molecules [82]. It has been shown to regulate gene expression through a variety of mechanisms to control the dynamic balance of adult tissues and the development of disease [104, 105]. During this period, related proteins, RNA, and genomic DNA are needed to synergistically regulate the gene expression. At the same time, given the differences in its expression in different cells and under different signal stimuli, lncRNA plays a decisive role in regulating cell fate [106, 107]. Recent studies have reported a regulatory effect of lncRNA on the functions of keratinocytes and epidermal cells in the skin [108]. Abnormally high expression of lncRNA in psoriatic skin has been observed by detecting lncRNA expression in normal and diseased skins, indicating that these lncRNAs may be associated with the underlying pathogenesis of the disease [109, 110]. Moreover, intriguingly, these differentially expressed lncRNAs have shown a synchronous expression with genes involved in the regulation of epidermal differentiation, which might be correlated [39].

In addition, many studies have detected the differential expression of lncRNAs between early-passage and late-passage dermal papilla, with the latter losing the ability to induce hair growth in the body. A total of 1683 lncRNAs showed an upregulated expression and the expression of 1773 lncRNAs decreased in early-passage dermal papilla compared with that in late-passage dermal papilla [111]. Similarly, in the study of skin aging, 151 lncRNAs were found to be differentially expressed between young and aging skins [112]. Although most of the aforementioned studies focused on the differential expression of many lncRNAs, these differentially expressed lncRNAs were actually associated with the proliferation and differentiation of epidermal cells [113].

The antidifferentiated lncRNA (ANCR) is abnormally highly expressed in epidermal progenitor cells and downregulated during differentiation [114]. The specific knockout of ANCR gradually results in an increase in the expression of transcription factors that promote differentiation, such as Grainyhead-like 3 (GRHL3), zinc finger protein 750 (ZNF750), positive regulatory domain 1 (PRDM1), and Kruppel-like factor 4 (KLF4), thus leading to the premature differentiation of the epidermis [115, 116]. In contrast, during epidermal differentiation, the expression of lncRNA (TINCR), which induces terminal differentiation, is abnormally elevated [115]. TINCR regulates epidermal differentiation by binding to Staufen 1 to form a complex that increases the stability of mRNAs promoting differentiation (e.g., KRT80, MAF, and MAFB) [115].

The findings indicated that differentially expressed lncRNAs were involved in the regulation of epidermal cell proliferation and differentiation by regulating related transcription factors or increasing the stability of related mRNAs. This confirmed that lncRNA regulated the biological activities of EpiSCs.

## 4. Clinical Application of EpiSCs

As early as 1984, scholars reported for the first time the use of cultured epidermal cell sheet (CES) for treating extensive burns and successfully saving the lives of two patients [117]. Currently, a consensus has been reached worldwide that transplanting CES into patients with extensive burns can increase their survival rate [118] because CES is rich in EpiSCs, which can undergo proliferation and differentiation after transplanting into wounds, thus achieving epidermal tissue regeneration [119]. In addition, in chronic trauma, the regeneration of epidermal tissues is difficult due to the small number of EpiSCs in local tissues and their depletion under continuous blood circulation [120], which undoubtedly aggravates the wound. Therefore, the inoculation of EpiSCs cultured in a carrier matrix compatible with ECM in chronic wounds not only effectively increases the number of EpiSCs but also provides the necessary matrix components for repairing wounds. In addition, EpiSCs located in the bulge of the outer sheath of hair follicles can not only migrate to the epidermis but also differentiate into sweat gland cells (involved in the formation of the sweat gland) and hair stromal cells (involved in hair regeneration). Some scholars used EpiSCs to treat severe skin burns, showing that the wound skin after repair had structures of the sweat gland and hair follicle tissue. Scarless healing was achieved through tissue regeneration in the aforementioned tissue defects, which is undoubtedly an important research direction.

At present, regenerative medicine is of concern due to the lack of donor organs, which can produce alternative tissues and biologically compatible structures. EpiSCs can be used as an important source of cells for replacing and repairing epithelial tissues. Hence, they have important clinical values in the regeneration treatment of other epithelial tissues. In fact, after the EpiSCs are separated from the body tissue and away from their intrinsic biological microenvironment, they exhibit the capacity far beyond the normal state, which has been demonstrated by the ability to differentiate into three embryonic germ layers after injection of EpiSCs into mouse embryos [121].

Epidermolysis bullosa (EB) is a serious skin disease caused by mutations of genes involved in the regulation of the adhesion of basal epithelial cells to the basal layer. The researchers conducted the corresponding lentivirus transformation of EpiSCs from the palms, which were reinserted into the thigh, in patients with EB having a mutation in the laminin-5 gene [122]. One year later, it was clinically observed that the laminin-5 synthesis in the transplanted region was still at a normal level, and the adhesion ability of the epidermal cells in the transplanted region was also normal, indicating that EpiSCs had therapeutic effects on EB and stable vitiligo [123, 124].

Vitiligo is a disease caused by the loss of melanocytes, while stable vitiligo refers to the stage when this disease enters a long-term resting period. As early as in 1992, the researchers attempted to transplant cultured keratinocytes and melanocytes to treat stable vitiligo [124]. Several recent studies have reported the use of EpiSCs to obtain CES and combination treatment with melanocytes [125, 126]. Studies have shown that the combination treatment with CES in the same patient is similar to traditional treatment in terms of efficacy [127]; after the treatment with CES, the transplant has a good connection with the skin in the recipient region, good color matching, and no scars [126]. In addition, some scholars have recently discovered that the use of CES alone can treat patients with localized vitiligo [128].

Limbal stem cell deficiency (LSCD) is a disease resulting from the damage or loss of limbal stem cells that maintain the homeostasis of corneal epithelial tissues. Recent studies have found many similarities between the cornea and cutaneous epithelial tissues, including typical stratified epithelial morphology and expression of p63. Related reports have suggested that, in animal models, the transplantation of EpiSCs can treat LSCD with a cure rate of 80% [129]. This can be attributed to the transformed EpiSCs with a degree of plasticity and regenerative capacity, which can be regenerated into the corneal epithelium and clear cornea in the LSCD animal model.

EpiSCs have long been considered seed cells for wound repair, and their role in wound repair has been confirmed in numerous laboratory studies. However, reports on the clinical trials of EpiSCs in wound repair are lacking. In the epidermal damage associated with some metabolic diseases, EpiSCs are used as vectors of gene therapy to correct congenital skin damage through genetic modification. Currently, clinical studies on the application of EpiSCs are available on the official website of the clinical trial (http://clinicaltrials/gov/). Using “epidermal stem cells” as the keyword, two ongoing clinical studies have been searched, both of which applied by the Paracelsus Medical University Dermatology Institute in Austria, besides evaluation studies of the safety and effectiveness of the transplantation of the autologous cultured and genetically modified ESC epidermis into patients with junctional epidermolysis bullosa and recessive dystrophic epidermolysis bullosa. Although the trials are only in the initial stages, the feasibility of EpiSCs for clinical transformation and application was confirmed. At present, tissue-engineered EpiSCs have been used as a potential treatment, but their long-term efficacy and related clinical trials still need further investigation. The success of laboratory applications shows the application prospects of EpiSCs in various clinical scenarios.
